# Longitudinal Patterns in Clinical and Imaging Measurements Predict Residual Survival in Glioblastoma Patients

**DOI:** 10.1038/s41598-018-32397-z

**Published:** 2018-09-26

**Authors:** Nova F. Smedley, Benjamin M. Ellingson, Timothy F. Cloughesy, William Hsu

**Affiliations:** 1 Department of Bioengineering, Henry Samueli School of Engineering, University of California Los Angeles, Los Angeles, CA USA; 2 Medical Imaging Informatics, Department of Radiological Sciences, David Geffen School of Medicine, University of California Los Angeles, Los Angeles, CA USA; 3 UCLA Brain Tumor Imaging Laboratory, Center for Computer Vision and Imaging Biomarkers, Department of Radiological Sciences, David Geffen School of Medicine, University of California Los Angeles, Los Angeles, CA USA; 4 UCLA Neuro-Oncology Program, David Geffen School of Medicine, University of California Los Angeles, Los Angeles, CA USA; 5 Department of Neurology, David Geffen School of Medicine, University of California Los Angeles, Los Angeles, CA USA

## Abstract

The growing amount of longitudinal data for a large population of patients has necessitated the application of algorithms that can discover patterns to inform patient management. This study demonstrates how temporal patterns generated from a combination of clinical and imaging measurements improve residual survival prediction in glioblastoma patients. Temporal patterns were identified with sequential pattern mining using data from 304 patients. Along with patient covariates, the patterns were incorporated as features in logistic regression models to predict 2-, 6-, or 9-month residual survival at each visit. The modeling approach that included temporal patterns achieved test performances of 0.820, 0.785, and 0.783 area under the receiver operating characteristic curve for predicting 2-, 6-, and 9-month residual survival, respectively. This approach significantly outperformed models that used tumor volume alone (p < 0.001) or tumor volume combined with patient covariates (p < 0.001) in training. Temporal patterns involving an increase in tumor volume above 122 mm^3^/day, a decrease in KPS across multiple visits, moderate neurologic symptoms, and worsening overall neurologic function suggested lower residual survival. These patterns are readily interpretable and found to be consistent with known prognostic indicators, suggesting they can provide early indicators to clinicians of changes in patient state and inform management decisions.

## Introduction

Glioblastoma (GBM) is an aggressive neoplasm associated with poor prognosis and cognitive decline^1^. The mean survival is 14.6 months, and 90% of patients experience tumor recurrence^2–4^. As such, patients are routinely followed up to evaluate health changes and to assess the quality of life. However, predicting prognosis in GBM has been a longstanding challenge. Each tumor is biologically heterogeneous; neurologic symptoms can fluctuate; pseudo-progression and tumor progression features overlap; and other complications can differentially affect patients^1,5^. Therefore, the ability to automatically flag individuals with early signs of poor prognosis would benefit clinicians who must decide if treatment strategies should be initiated or changed.

While studies have identified prognostic clinical, imaging, and molecular traits^5–8^, they are typically analyzed at a single time point or based on observations not routinely available in the clinic (e.g., multimodal genomic data). The importance of capturing the longitudinal evolution of a disease—the *disease trajectory*—has been recognized. The Levin criteria assess a combination of qualitative changes in computed tomography or magnetic resonance (MR) images and neurologic function compared to prior visits^9^. Changes in tumor volume combined with stable neurological scores and the use of corticosteroids are important factors in evaluating treatment response^10–12^. While changes are documented, recognizing the patterns’ significance is left to the clinician.

Machine learning algorithms are well suited for discovering predictive patterns from large, complex data. One effort involved discovering sequential events in clinical workflow logs to expose medical behaviors that could improve patient care^13^. Sequential patterns have also been applied to patients’ medical data to predict outcomes such as thrombocytopenia^14^, cardiovascular-related diagonses^14^, and diabetes medication prescriptions^15^. Other investigators used sequential pattern mining to identify treatment pathways in GBM patients that were predictive of one-year overall survival^16^ using data from The Cancer Genome Atlas (TCGA); they focused on drug treatment patterns and multimodal molecular traits. However, the TCGA dataset is inherently limited in temporal resolution, has inconsistently collected multicenter institutional data that provides a varied amount of information, and does not use imaging information.

We developed and evaluated a method to mine longitudinal patterns, termed *temporal patterns*, from clinical and imaging data to determine residual survival at an individual patient encounter. Residual survival is defined as the remaining number of days until death for a patient at a given clinical visit. We applied machine learning to select temporal patterns that were predictive of 2-, 6-, or 9-month residual survival. Our objective was to eventually develop a decision support tool that aids clinicians when assessing patients to identify patterns that predict death.

## Materials and Methods

### Patients and Data Collection

We included patients with pathologically confirmed GBM that underwent standard chemoradiotherapy (i.e., radiotherapy with concomitant temozolomide^2^) in whom complete neurological and imaging data available December 1999 – May 2015. Each visit was defined by one or more events. An event was a surgical procedure, radiation treatment, neurological exam, or tumor measurement (Supplementary Fig. S3). Data was cleaned by removing 762 events with missing information and 14 events with invalid values (e.g., typographical errors). The final cohort included 304 patients (Table 1) with 7078 visits and 24989 events. Patients had an average of 695.7 follow-up days, 28.4 days between visits, and 23.3 visits (Supplementary Fig. S2). The archival and research use of patient’s clinical, biological, and image data were approved by UCLA Institutional Review Board (Medical IRB2). All patients signed the institutional review board–approved informed consent. All methods were carried out in accordance with relevant guidelines and regulations. All experimental protocols were approved by the named institutional committee.

**Table 1:** Patient characteristics (n = 304).

Category	Trait	N	%
Overall Survival	Mean	721.2	—
	Standard deviation	534.1	—
Status	Deceased	257	84.5%
	Alive	47	15.5%
Initial age	Mean	54.6	—
	Standard deviation	11.9	—
Gender	Male	188	61.8%
	Female	116	38.2%
Ethnicity	White	217	71.4%
	Hispanic	24	7.9%
	Asian	17	5.6%
	Middle eastern	4	1.3%
	Black	2	0.7%
	Other	15	4.9%
	Unknown	25	8.2%
*MGMT* promoter status	Methylated	103	33.9%
	Unmethylated	201	66.1%
Initial tumor laterality	Right	162	53.3%
	Left	149	49.0%
Initial tumor location	Frontal lobe	114	37.5%
	Temporal lobe	91	29.9%
	Parietal lobe	76	25.0%
	Occipital lobe	18	5.9%
	Thalamus	8	2.6%
	Corpus callosum	4	1.3%
	Cerebellum	1	0.3%
	Pineal gland	1	0.3%
	Midbrain	1	0.3%

There were seven bilateral tumors and eleven that spanned two locations.

### Clinical Data

Patient covariates that were considered static without change over time, included sex, race/ethnicity, *MGMT* promoter methylation status, and initial tumor location and laterality; dynamic variables subject to change during follow-up included age (the only dynamic patient covariate), interventions, neurologic evaluations, and tumor volumes (Table 2). For radiation treatment and chemotherapy, only initiation dates were considered. Neurologic evaluations included Karnofsky performance status (KPS), neurologic function, mental status, and overall neurologic function (based on the Levin criteria). Discretization of continuous variables was necessary for subsequent steps in machine learning (see Supplementary Methods).

**Table 2:** Variables used by sequential pattern mining to generate the temporal patterns.

Variable	State	Definition
Surgery	Occurred	Patient underwent a surgical procedure.
Radiation	Occurred	Patient began receiving radiotherapy.
KPS	Initial	First KPS observed
	Decreased	KPS has dropped but remains above 60
	Significant decrease	KPS has dropped to or below 60
	Increase	KPS was above 60 and has increased
	Significant increase	KPS was at or below 60 and has increased
	Unchanged	All other KPS values
Mental status	0	Normal function
	1	Minor mental confusion
	2	Gross confusion but awake
Neurological function	0	No symptoms, fully active at home/work without assistance
	1	Minor symptoms, fully active at home/work without assistance
	2	Moderate symptoms, fully active at home/work without assistance
	3	Moderate symptoms, less than fully active at home/work without assistance
	4	Severe symptoms, totally inactive requiring complete assistance, unable to work
Overall neurological status	+2	Definitely better compare to prior observation
	+1	Possibly better compared to prior observation
	0	Unchanged compared to prior observation
	–1	Possibly worse compared to prior observation
	–2	Definitely worse compared to prior observation
Tumor volume		
Volume (V)	*	Tumor volume (mm^3^)
Baseline volume	*	First tumor volume (mm^3^) measured after completion of chemoradiation
Rate change	*	V_2_ − V_1_/D_2_ − D_1_ (mm^3^/day); between two sequential visits
Percent change	*	100% × (V_2_ − V_1_)/V_1_ (%); between two sequential visits
Response criteria	Complete	Resolution of all enhancement
	Partial	≥65% decrease in volume
	Progression	≤40% increase in volume
	Stable	All others

The *denotes continuous variable which were discretized into ten states with equal frequency. D = days since baseline.

### MR Imaging and Post-processing

Anatomic MR images were acquired for all patients using a 1.5 T or 3 T clinical MR scanner with pulse sequences supplied by their respective manufacturers and according to routine care protocols. Standard anatomic images were obtained with the axial T1-weighted fast spin-echo sequence or magnetization-prepared rapid acquisition gradient-echo (MPRAGE) sequence (repetition time = 400–3209 msec; echo time = 3.6–21.9 msec; inversion time = 0–1238 msec; slice thickness = 1–6.5 mm; intersection gap = 0–2.5 mm; number of averages = 1–2; matrix size = 176–512 × 256–512; and field of view = 24–25.6 cm).

Parameter-matched T1-weighted images enhanced with gadopentetate dimeglumine (Magnevist; Berlex), 0.1 mmol/kg, were acquired shortly after contrast material injection. Contrast-enhanced T1-weighted subtraction maps were created with techniques previously used to define contrast-enhancing tumor volume in the presence of changes in vascular permeability^17,18^. Estimates of tumor volume (in mm^3^) included areas of contrast enhancement on T1 subtraction maps plus any areas of central necrosis.

In addition to examining actual tumor volumes (in mm^3^), we explored four other representations of tumor volume: baseline volume, the rate of change, percent change, and the volumetric-equivalent RANO response criteria^12,19–21^, all calculated by comparing the current and previous tumor measurements (Table 2).

### Temporal Patterns

**Figure 1:**
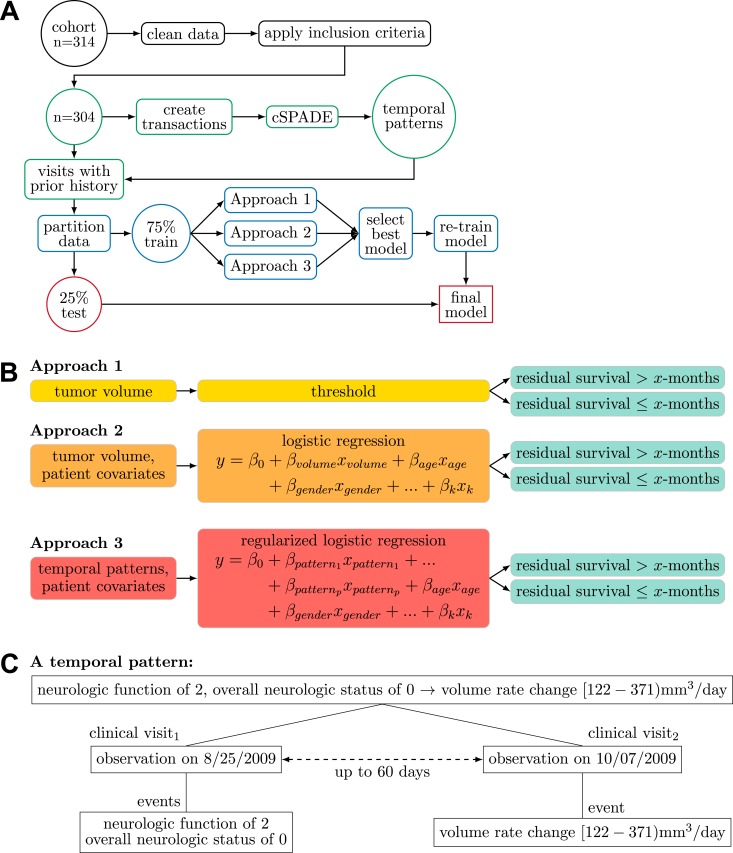
(**A**) An overview of the study using data mining and machine learning to model residual survival. (**B**) The three approaches used to predict residual survival given tumor volume, *k* patient covariates and *p* temporal patterns. (**C**) An example of a temporal pattern mined from longitudinal patient data.

Temporal patterns were generated using a sequential pattern mining algorithm called cSPADE^22,23^. Figure 1A illustrates the overall process for identifying temporal patterns. cSPADE used all variables in Table 2. Each event was considered a single “item”. Each clinical visit was considered a “transaction” (i.e., a collection of items). A patient’s history can be seen as a distinct ordering of transactions called a “sequence.”

cSPADE requires the following set of input parameters to constrain the types of patterns discovered by the algorithm. Minimum support requires a pattern to be observed in at least a certain percentage of patients. For example, the event “KPS significantly decreased” was observed in 133 of 304 patients and achieved a support of 0.44 (44% of patients). If minimum support were set to 0.2, the event would be considered a frequent sequence. Maximum gap limits the number of days between any two consecutive visits in a pattern and was set based on the typical follow-up schedule for patients. Maximum length specifies the maximum number of visits in an identified pattern. Maximum size defines the maximum number of events that would be considered for any given visit. A pool of 1009 parameter combinations was explored to identify optimal values (see Supplementary Materials).

### Modeling

#### Model Generation

A logistic regression model was formulated to estimate each of three prognostic tasks—2-, 6-, or 9-month residual survival—given events observed in recent clinical visits. Both patient covariates and temporal patterns were used as features in the logistic model. To train a model, labels were assigned to each clinical visit. For example, to train the 2-month prognostic model, visits were labeled as “residual survival ≤ 2 months” if observed within two months of death; otherwise, the visit was labeled as “residual survival > 2 months.” Temporal patterns were converted into binary features to indicate—with a value of 0 (not observed) or 1 (observed)—whether the sequence was observed at each clinical visit. For example, in Fig. 1C, the pattern was observed if at a current visit the patient had a rate of volume change [122–371) mm^3^/day preceded by a visit within 60 days where the neurologic function was 2 (moderate symptoms), and overall neurologic function was 0 (unchanged compared to prior observation). Given a large number of possible temporal patterns, the number of features included in the regression model was limited using the least absolute shrinkage and selection operator (LASSO)^24^.

Many logistic models were trained; each used a set of temporal patterns generated from each combination of cSPADE parameters. Visits with insufficient prior observations were not classified. For example, if the maximum length was set to 3 visits and the maximum gap was set to 60 days, cSPADE would search for patterns with maximally 3 clinical visits and maximally 60 days between any two consecutive visits in the set of 3. To definitively determine whether these patterns occurred, a minimum of 120 days of documented prior history was required.

#### Model Training

Patients were randomly split into 75% training and 25% testing sets. The following two-step splitting method was used to ensure a balanced distribution of clinical visits and gender in each set. Patients were first separated based on whether their overall survival was lower than the mean overall survival and resulted in two subgroups. For each survival subgroup, patients were split into 75% training and 25% testing partitions while conserving the proportion of males to females in each part. The training and testing partitions of each survival subgroup were merged to create the overall training and testing sets. Thus, each patient’s set of clinical visits were either used for training or testing, but not both. Observed proportions of “residual survival > x-months” and “residual survival ≤ x-months” classes in the total dataset were relatively maintained across the training and testing sets. The testing set was set aside for a final evaluation of the fully trained models.

With the training partition, 10-fold cross-validation was used to identify the model parameters with the highest discrimination. The 10-fold split was patient-based, where patients in the validation fold did not have clinical visits in the remaining training folds. Model performance was measured using the area under the receiver operating characteristic curve (AUC) and the area under the precision-recall curve (average precision). There were more observations categorized as residual survival > x-months than residual survival ≤ x-months by a ratio of 19:1, 3.4:1, and 2:1 for predicting 2-, 6-, and 9-month survival, respectively. To address this data imbalance, down-sampling was performed during training by randomly selecting a subset of the larger class. This process created a balanced class ratio for training. The 10-fold cross-validation procedure was performed three times with different seeds for random fold splits, and the performance was averaged. The model with the highest averaged cross-validated AUC was re-trained on the entire training set to derive the final coefficients. The final model was then evaluated using the held-out testing set. This process was repeated for each model predicting 2-, 6-, and 9-month residual survival.

#### Evaluation

We compared three different approaches to predict residual survival at each clinical visit (see Fig. 1B). In the first approach, only tumor volume information was used. Visits, where patients had tumor volume measurements above a specified threshold, were predicted as residual survival ≤ x-months. AUC was evaluated across all possible volume thresholds. In the second approach, we built logistic regression models using tumor volume and patient covariates as features. Lastly, we developed another set of logistic regression models that used patient covariates and temporal patterns as features.

The top performer was taken from each approach and their AUC on the training partition was used for pair-wise comparisons. The 95% confidence intervals (CI) of individual training AUCs were obtained by bootstrapping^25^ with 2000 stratified replicates. Statistical differences between training AUCs of these approaches were evaluated with bootstrap tests^25^ using 2000 replicates. Univariate analyses were performed for the top ten patterns for residual survival ≤ x-months and residual survival > x-months. Patterns were ranked based on their adjusted odds ratios from the final model. Fisher’s exact test was used to compute the univariate odds ratio, its 95% CI, and whether it significantly differed from an odds ratio of 1 for each pattern. An α level of 0.05 was considered significant for all statistical tests.

## Results

### Predicting Survival with Tumor Volume Measurements

Of 304 patients, 289 had one or more tumor volume measurements after completion of initial chemoradiotherapy, resulting in a total of 3917 usable measurements. Alternative representations of tumor volume measurements (continuous volume, baseline volume, rate change, and percent change; see Table 2) were each evaluated individually. Among these, continuous volume measured in mm^3^ achieved the highest performance in the training partition with AUCs of 0.769 (95% CI: 0.726–0.810), 0.750 (95% CI: 0.728–0.772), and 0.745 (95% CI: 0.726–0.764) for predicting 2-, 6-, and 9-month survival, respectively (Supplementary Figs S4–S5).

### Predicting Survival with Discretized Tumor Volume and Patient Covariates

Models incorporating tumor volume measured in mm^3^ or discretized rate change had similar performances and were the top-performing models in predicting 2-, 6-, and 9-month survival (Supplementary Fig. S6). The addition of patient covariates had little or no improvement over the first approach defined earlier for tumor volumes in mm^3^ and rate change. In repeated cross-validation, models using covariates only improved if the prediction AUC was around or below 0.5 (i.e., no better or worse than random guesses) with using just tumor volume information. The use of either the volumetric RANO response criteria, percent volume change, or baseline volume consistently had lower classification performances for 2-, 6-, and 9-month survival with respective average AUCs in the ranges of 0.531–0.634, 0.516–0.620, and 0.562–0.609. Continuous volume measures in 2- and 6-month survival had the highest averaged AUC, while discretized rate change was the best predictor in the 9-month survival model. These models had an AUC of 0.779 (95% CI: 0.739–0.817), 0.750 (95% CI: 0.724–0.774), and 0.762 (95% CI: 0.741–0.782), respectively in the training partition.

### Predicting Survival with Temporal Patterns and Patient Covariates

Supplementary Fig. S7 visualizes the classification performance across the top 15 cSPADE parameter combinations out of 1009 explored during repeated cross-validation. Among the different tumor volume measurements used in creating temporal patterns, discretized rate change had consistently higher performance in all three survival prediction tasks. Subsequently, these temporal patterns achieved an AUC of 0.879 (95% CI: 0.858–0.897), 0.868 (95% CI: 0.856–0.880) and 0.854 (95% CI: 0.842–0.866), respectively for 2, 6, and 9 months in the training partition.

The top-performing model for 2-month survival had 41 variables in the logistic regression model. These variables were selected from a pool of patient covariates and the 3758 temporal patterns generated from a minimum support of 0.3, a maximum gap of 60 days between visits, a maximum length of 3 visits, and a maximum size of 3 events per visit. For this cSPADE combination, there were 5166 visits available for modeling. This approach outperformed the top performers from using tumor volume alone (AUC: 0.879 vs. 0.769; p < 0.001) and tumor volume with patient covariates (AUC: 0.879 vs. 0.777; p < 0.001) for predicting 2-month survival in the training partition.

**Figure 2:**
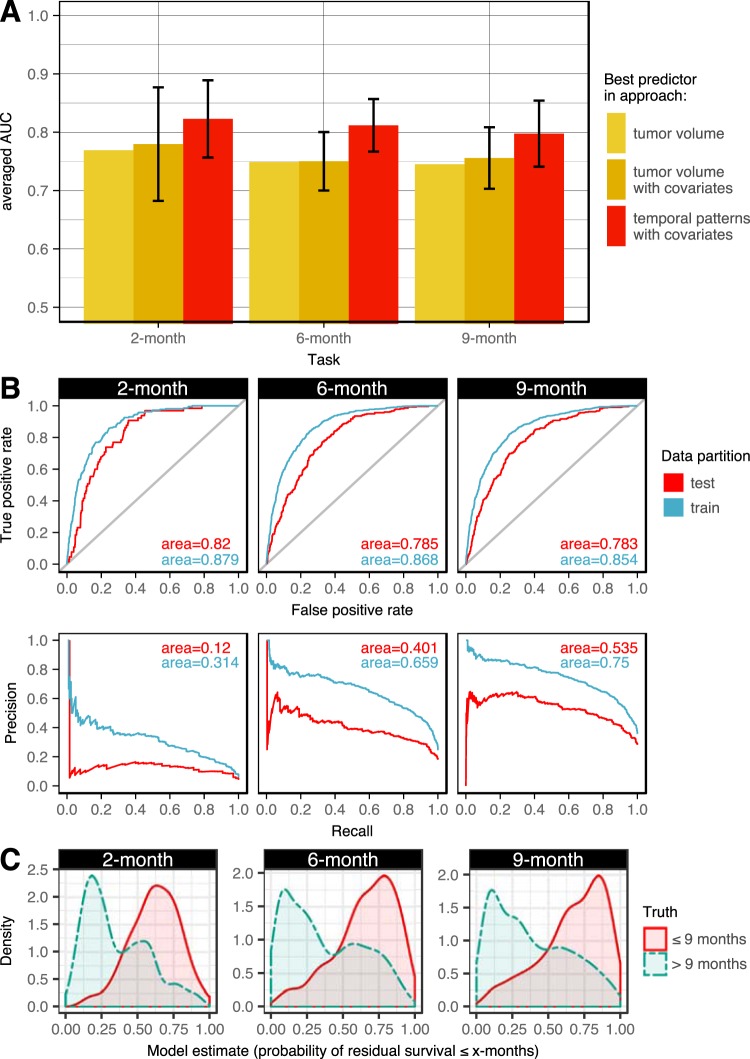
(**A**) The training performance of the best performers from each approach. Error bars are AUC standard deviations if cross validation was used, where performance scores were averaged across folds. (**B**) The performance of the selected models after fitting on the entire training partition. (**C**) Density plots showing the models’ predictions compared to the ground truth in test cases. Complete separation (no overlap) of the two class distributions signifies a model with perfect classification.

Similarly, the top models for 6 and 9 months used 115 and 94 variables, respectively. The top 6-month survival model selected from a pool of patient covariates and 5944 temporal patterns generated from a support of 0.25, a gap of 60 days, a length of 3 visits, and a size of 4 events as parameters. The top 9-month model considered 4420 patterns generated from a different support of 0.30, but the same gap, length, and size from the top 6-month model. Since the gap and length parameters are the same among the top models for each 2-, 6- and 9-month prediction, all three models had the same number of visits left for modeling. The 6-month model outperformed the top performers that used tumor volume alone (AUC: 0.868 vs. 0.750; p < 0.001) and tumor volume with patient covariates (AUC: 0.868 vs. 0.745; p < 0.001). The 9-month model also outperformed approaches using tumor volume alone (AUC: 0.854 vs. 0.747; p < 0.001) and tumor volume with patient covariates (AUC: 0.854 vs. 0.761; p < 0.001). This approach produced models with the highest performance for all three prediction tasks (Fig. 2A) and was selected as the final models for testing evaluation.

### Performance of the Final Model

The final models had testing AUCs of 0.820 (95% CI: 0.777–0.859), 0.785 (95% CI: 0.757–0.811), and 0.783 (95% CI: 0.759–0.807) for 2-, 6-, and 9-month survival, respectively (Fig. 2B). The 9-month model had the highest average precision in testing. Of the many variables that were ultimately selected by each of these logistic regression models, Tables 3–5 reports the highest contributing variables, ranked by their adjusted odds ratio (OR) for predicting each class, where an OR > 1 indicates the model’s belief that the patient will not survive the upcoming months. Conversely, an OR < 1 increases the model’s belief that the patient will survive the upcoming months.

**Table 3:** The top ten patterns used to predict residual survival of ≤2-months or >2-months (*italicized*).

S	Adj. OR	Univariate OR [95% CI]	Pattern
0.45	2.65	11.8 [8.34, 16.7]*	KPS significantly decreased
0.42	2.24	3.17 [2.08, 4.72]*	overall neurologic status of 0 → KPS unchanged → overall neurologic status of −1
0.31	2.21	3.13 [2.06, 4.63]*	volume rate change [371–5878] → KPS unchanged
0.46	2.07	7.18 [5.19, 9.85]*	volume rate change [371–5878]
0.31	1.92	3.59 [2.10, 5.88]*	neurologic function of 1, mental status of 0 → overall neurologic status of −1
0.31	1.54	2.48 [1.54, 3.87]*	overall neurologic status of 0, KPS unchanged → KPS decreased → KPS unchanged
—	1.51	1.96 [1.51, 2.55]*	right-sided tumor
0.37	1.45	0.76 [0.37, 1.41]	volume rate change [−167– −46) → KPS unchanged → KPS unchanged
0.33	1.32	1.73 [1.10, 2.64]^†^	overall neurologic status of −1 → KPS unchanged → KPS unchanged
0.52	1.28	2.11 [1.40, 3.09]*	KPS decreased → KPS unchanged
0.31	*0*.*30*	*0*.*00 [0*.*00*, *0*.21]*	volume rate change [−4–0.17)
*0*.*30*	*0*.*46*	*0*.*10 [0*.*01*, *0*.35]*	volume rate change [7.7–40) → KPS unchanged → KPS unchanged
*0*.*32*	*0*.*46*	*0*.*11 [0*.*00*, *0*.64]*	overall neurologic status of 0 → overall neurologic status of 0 → volume rate change [7.7–40)
*0*.*40*	*0*.*47*	*0*.*06 [0*.*00*, *0*.36]*	volume rate change [−13–−4)
*0*.*38*	*0*.*52*	*0*.*16 [0*.*00*, *0*.*90]*^*†*^	KPS increased
*0*.*78*	*0*.*53*	*0*.*19 [0*.*14*, *0*.25]*	KPS unchanged, mental status of 0
*0*.*72*	*0*.*60*	*0*.*24 [0*.*16*, 034]*	neurologic function of 1
*0*.*33*	*0*.*65*	*0*.*06 [0*.*00*, *0*.33]*	volume rate change [0.17–7.7)
*0*.*86*	*0*.*69*	*0*.*16 [0*.*11*, *0*.22]*	overall neurologic status of 0, KPS unchanged
*0*.*34*	*0*.*70*	*0*.*16 [0*.*07*, *0*.31]*	neurologic function of 0, overall neurologic status of 0

Patterns were ranked by adjusted odds ratio (adj. OR) from regression modeled with 5825 visits (see Supplementary Methods). S = support, the proportion of patients with the pattern. ^†^p < 0.05; *p < 0.001.

**Table 4:** The top ten patterns used to predict residual survival of ≤6-months or >6-months (*italicized*).

S	Adj. OR	Univariate OR [95% CI]	Pattern
0.46	2.65	7.00 [5.37, 9.16]*	volume rate change [371–5878]
0.30	2.28	2.36 [1.79, 3.10]*	volume rate change [122–371) → overall neurologic status of 0
—	1.95	1.67 [1.46, 1.91]*	right-sided tumor
0.49	1.94	3.19 [2.45, 4.14]*	volume rate change [122–371)
0.25	1.92	4.01 [2.74, 5.87]*	volume rate change [371–5878] → volume rate change [−26,700–−167)
0.26	1.76	2.07 [1.53, 2.79]*	overall neurologic status of −1, mental status of 0 → mental status of 0 → KPS unchanged
0.39	1.72	4.02 [3.22, 5.03]*	neurologic function of 3
0.31	1.72	2.90 [2.21, 3.81]*	volume rate change [371–5878]
0.27	1.67	2.91 [2.17, 3.88]*	mental status of 0 → overall neurologic status of −1, KPS unchanged
0.28	1.61	2.51 [1.71, 3.65]*	overall neurologic status of 0, KPS unchanged → overall neurologic status of 0, KPS unchanged → volume rate change [122–371)
0.28	*0*.*23*	0.29 [0.10, 0.66]*	overall neurologic status of 0 → KPS increased
*0*.*31*	*0*.*24*	0.14 [0.07, 0.25]*	volume rate change [−4–0.17)
*0*.*34*	*0*.*28*	0.00 [0.00, 0.83]^†^	neurologic function of 1, initial KPS → mental status of 0 → mental status of 0
*0*.*47*	*0*.*44*	*0*.*00 [0*.*00*, *2*.*01]*	initial KPS → neurologic function of 1
*0*.*78*	*0*.*49*	*0*.*27 [0*.*23*, *0*.31]*	KPS unchanged, mental status of 0
*0*.*33*	*0*.*54*	*0*.*22 [0*.*13*, *0*.36]*	volume rate change [0.17–7.7)
*0*.*28*	*0*.*55*	*1*.*00 [0*.*72*, *1*.*36]*	neurologic function of 0
*0*.*38*	*0*.*55*	*0*.*16 [0*.*11*, *0*.22]*	KPS decreased → overall neurologic status of 0, mental status of 0 → mental status of 0
*0*.*92*	*0*.*57*	*0*.*30 [0*.*26*, *0*.34]*	KPS unchanged
*0*.*34*	*0*.*59*	*0*.*13 [0*.*09*, *0*.18]*	neurologic function of 0, overall neurologic status of 0

Patterns were ranked by adjusted odds ratio (adj. OR) from regression modeled with 5155 visits. S = support, the proportion of patients with the pattern. ^†^p < 0.05, *p < 0.001.

**Table 5:** The top ten patterns used to predict residual survival of ≤9-months or >9-months (*italicized*).

S	Adj. OR	Univariate OR [95% CI]	Pattern
0.46	4.97	7.79 [5.78, 10.6]*	volume rate change [371–5878]
0.33	2.57	2.82 [1.87, 4.28]*	Surgery
0.49	2.35	3.67 [2.82, 4.81]*	volume rate change [122–371)
0.35	2.18	2.77 [2.19, 3.50]*	volume rate change [122–371) → KPS unchanged
0.31	2.03	2.77 [2.11, 3.63]*	volume rate change [371–5878] → KPS unchanged
—	2.00	1.61 [1.43. 1.81]*	right-sided tumor
0.33	1.82	7.04 [4.65, 10.9]*	KPS unchanged → KPS significantly decreased
0.31	1.81	3.99 [2.73, 5.91]*	mental status of 0 → overall neurologic status of −1, KPS unchanged
0.33	1.79	2.06 [1.46, 2.61]*	overall neurologic status of −1 → KPS unchanged → KPS unchanged
0.32	1.76	3.44 [2.49, 4.77]*	KPS unchanged → KPS unchanged, mental status of 0 → overall neurologic status of −1
0.57	*0*.*25*	*0*.*00 [0*.*00*, *0*.89]*	initial KPS → KPS unchanged, mental status of 0
*0*.*31*	*0*.*36*	*0*.*12 [0*.*00*, *0*.*80]*^*†*^	neurologic function of 1, initial KPS → neurologic function of 1, KPS unchanged → neurologic function of 1
*0*.*31*	*0*.*38*	*0*.*16 [0*.*10*, *0*.25]*	volume rate change [−4–0.17)
*0*.*34*	*0*.*39*	*0*.*13 [0*.*10*, *0*.18]*	volume rate change [−13–−4)
*0*.*34*	*0*.*44*	*0*.*00 [0*.*00*, *0*.*48]*^*†*^	neurologic function of 1, initial KPS → mental status of 0 → mental status of 0
*0*.*33*	*0*.*51*	*0*.*22 [0*.*14*, *0*.32]*	volume rate change [0.17–7.7)
*0*.*40*	*0*.*62*	*0*.*33 [0*.*23*, *0*.47]*	volume rate change [−13– −4)
*0*.*92*	*0*.*65*	*0*.*31 [0*.*28*, *0*.47]*	KPS unchanged
*0*.*38*	*0*.*69*	*0*.*17 [0*.*13*, *0*.22]*	neurologic function of 0
*0*.*60*	*0*.*77*	*0*.*42 [0*.*36*, *0*.48]*	neurologic function of 1, KPS unchanged, mental status of 0

Patterns were ranked by adjusted odds ratio (adj. OR) from regression modeled with 5166 visits. S = support, the proportion of patients with the pattern. ^†^p < 0.01, *p < 0.001.

The top patterns included every category of variables listed in Table 2. While neurologic evaluations were clinically related to each other, the correlation between exams showed non-overlapping information (see Supplementary Materials). Often, the rate of volume change was an event in patterns with adjusted ORs above 2. A majority of the top ten patterns were significant in univariate analysis, indicating that prominent patterns were selected into the models and provided complementary information for residual survival prediction. After adjusting for other temporal patterns and patient covariates, the top patterns for residual survival ≤ x-months generally had lower adjusted ORs than univariate ORs. Similarly, the models estimated higher ORs than univariate analyses for residual survival > x-months.

**Figure 3:**
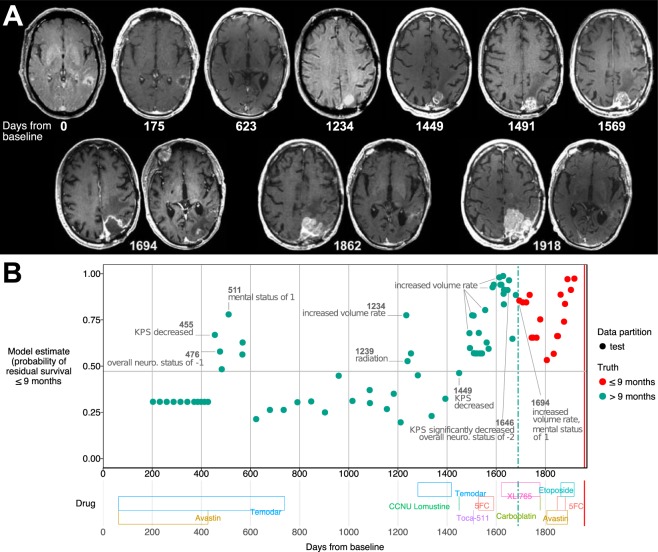
An example of a white male initially diagnosed in his 60′s. (**A**) MR imaging showing tumor at baseline, regrowth, and remote recurrence near end of life. (**B**) The 9-month residual survival model’s estimates given patient covariates and temporal patterns observed at each clinical visit. Gray horizontal line is a threshold (see Supplemental Methods) used for classifying residual survival from the predicted probabilities. The vertical green dash line is 9 months (270 days) from the vertical red line (day of death).

Figure 3 illustrates one patient’s disease trajectory, as estimated by the 9-month model. The patient received initial chemoradiotherapy and began routine clinical visits 78 days later. The model had a maximum length of 3 visits and a maximum gap of 60 days between visits; therefore, the first possible prediction occurred on day 203. The model accurately classified the patient’s status for a many of the observations. Temporal patterns occurring within 9 months of death were all correctly classified as residual survival ≤ 9-months. However, the model made the following key mistakes found across predictions tasks:

If a negative pattern was observed, the information can influence residual survival prediction in subsequent visits. For example, there was a KPS decrease on day 455 followed by slightly negative neurological scores on days 476, and 511, see Fig. 3. This sequence of events led the model to estimate a higher probability of residual survival ≤ 9-months. For visits 476 and 511, the model maintained a lower probability of residual survival due to the KPS decrease and not observing signs of good health. In reality, the patient likely had minor to moderate symptoms but recovered. Thus, the model misclassified the patient to have residual survival ≤ 9-months for several visits after 455.

If a patient had additional interventions, the model estimated lower residual survival. This is likely due to the frequent observation that patients in decline are given secondary treatments or switch treatment plans and such events occur towards the end of life. Thus, the model will misclassify a patient if additional treatment was given and it was effective, resulting in the patient having longer residual survival.

If a patient has series of tumor volume rate increases, the models have a high estimation of death in upcoming months. This was particularly true in Fig. 3, where the patient had a high probability of death for a year prior to the death date.

A detailed description of this patient case can be found in Supplementary Fig S8, and additionally, S9–S11 show other patient cases with varying degrees of classification accuracy.

Some patient covariates were included in the final models. Gender and *MGMT* promoter methylation were both selected into the 6- and 9-month models. The adjusted odds ratios were 1.37 and 1.16 for males, and 1.54 and 1.3 for unmethylated promoters, respectively, for the two models. The odds ratios are higher than 1.0, indicating males and unmethylation statuses had a slightly higher relative risk (for lower residual survival) than females and methylated statuses. Various age decades were also selected into the final models. Ages above 50 had contributed to 6- and 9- month death probability and age above 70 contributed the most to 2-month death probability. Tumor location was considered by all models; tumors located in the frontal lobe had ORs greater than 1, while parietal lobe and other locations had ORs smaller than 1. Lastly, patients with tumors on the right side a top-ranked feature in all three models. In general, the effect of covariates was small, as there were over 50 variables in the models and the top variables have greater or smaller ORs and subsequently has more effect on residual survival probability.

## Discussion

An unprecedented amount of clinical information can now be captured by clinicians for research purposes and by electronic medical records. However, clinicians are presented with the challenge of interpreting this data and identifying trends to inform individualized decision-making. Machine learning techniques can help identify patterns buried in longitudinal data. Here, we present an approach to detect recurring patterns in patient records that are predictive of prognosis. We demonstrate the potential for combining patient covariates with temporal patterns generated from changes in tumor volume and neurological scores to predict residual survival for GBM patients initially treated with standard chemoradiotherapy.

Our experiments underscore the importance of considering tumor volume as a key prognostic marker of survival, as it appears in many of the top-ranked patterns in all residual survival prediction tasks. For 2-month predictions, the pattern associated with about a 2-fold increase in death (adjusted odds ratio, adj. OR: 2.07) was tumor volume change above 371 mm^3^/day; this rate change translates to a tumor volume increase of up to 22.26 cm^3^ between two consecutive visits within 60 days. The highest contributor to death was a significant decline in KPS to below a score of 60. Also associated with a two-fold increase in death over survival were a sustained decrease in KPS (e.g., KPS decreased and remained unchanged in a subsequent visit) and an overall neurologic function of −1 (moderate decline) after a series of no symptom changes. Conversely, patients who were observed to have mild or no impairment in neurologic function had lowered odds of death. Other patterns that were associated with a 2-fold decrease in odds of death over survival include series of unchanging KPS, small changes in tumor volumes, and normal mental status.

The observation of shrinking tumors followed by unchanged KPS and neurologic function had a 1.45-fold relative increase in death over survival. While counterintuitive at first, these patterns may reflect patients that had an initial response to treatment, but the tumor recurred. A similar scenario was also found in the 6-month model, where an increasing volume rate increase was followed by shrinking tumor volume rates (response to secondary treatment) within 60 days of each other. However, these patients were likely to have a residual survival of fewer than 6 months (adj. OR: 1.92).

A key difference in the 6-month model, compared to the 2-month model, was that twice as many variables were selected into the model. Tumor volume rate increases above 122 mm^3^/day were often indicative of poorer 6-month residual survival, as were observations of patients that were less active, i.e., a neurological function of 3. Indicators of residual survival longer than 6-month, include small decreases in tumor volume rate (likely representative of successful drug therapy), and neutral/normal neurological evaluations. Unlike the 2-month model, this model had tolerance to a decreased in KPS if the patient was observed with normal or unchanged neurological exams (adj. OR: 0.55).

In the 9-month model, patterns of unchanged or asymptomatic clinical visits followed by a negative change were associated with some of the largest increased odds of death. For example, these temporal patterns included unchanged KPS followed by a significant decrease in KPS (adj. OR: 1.82). The event of an additional surgery was the second highest contributing feature to death in 9-months and was not observed in 2- or 6-month models. Patterns associated with improved OR for residual survival greater than 9 months included patients who were fully active without assistance at home or work and small changes in tumor volume. Lastly, ages ≥ 50, male, and unmethylated *MGMT* promoter status were selected features in the final models and had ORs ≥ 1, which is similar to other reports^3^. An unmethylated MGMT promoter had a higher relative risk for lower survival. This finding was consistent with current literature, where patients with an unmethylated MGMT promoter have shorter overall survival than patients with methylation as the former do not benefit from temozolomide (currently the most effective chemotherapeutic for these patients). The inclusion of covariates indicates complementary information to the combination of tumor volume and various neurologic examinations.

These results demonstrate the value of systematically and regularly capturing features to facilitate the training of predictive models. Particularly in GBM, outcomes such as recurrence, treatment efficacy, and survival are manually assessed at each clinical visit. In contrast, an algorithm could be applied in real-time to automatically detect patient decline via regularly updated patient observations. Timely actions such as increasing the follow-up frequency to noticing when treatment plans need to be adjusted could be vital for a disease with low overall survival. Building from our work, a clinical decision support system could be developed to visualize data trends (e.g., view patients with similar treatment pathways and tumor growth patterns) or model trends (e.g., estimated probabilities of patient outcomes). The system could incorporate this information into electronic health records to enable streamlined data collection to for analyses such as the work presented here.

There were several limitations of this study. We considered a limited number of variable types, where several provided some mutual information. We were unable to include IDH1 and IDH2 mutation information, as they have been found to be prognostic biomarkers in GBM. Likewise, there are other biomarkers, such as G-CIMP, hTERT, EGFRvIII that was not readily available for our study population, but potentially could be incorporated in future analyses. Furthermore, cSPADE was dependent on discrete variables and event frequencies; models were influenced by the discretization methods and the uniqueness of our patient cohort, impacting the models’ translation to other datasets. In future work, we will focus on temporal patterns that are indicative of chemotherapy failure; explore additional variables, such as more detailed imaging-based features; and use other statistical (e.g., Cox regression), probabilistic (e.g., Markov model), and machine learning–based models.

In summary, we demonstrate the value of using tumor volumetric measurements and temporal patterns in predicting patient prognosis at a clinical visit. We show that analyzing longitudinal patient data using sequential pattern mining yields temporal patterns that are intuitively meaningful and are prognostically useful. The models allow a more holistic, yet quantitative approach for assessing relatively short-term patient prognosis. The predictions are based on a window of time and subsequently, change as patient observations change. The outcome of our framework can be used as one indicator of how well a patient is doing and can facilitate clinical decisions about subsequent care, such as continued therapy versus palliative care.

## Electronic supplementary material

Supplemental Materials

## Data Availability

The dataset utilized for this study was extracted from a research database consisting of patients seen at our institution and has not been made publicly available due to the presence of protected health information (e.g., dates of follow-up to track sequence and days between observations).
