# Supplementary material for: Longitudinal Patterns in Clinical and Imaging Measurements Predict Residual Survival in Glioblastoma Patients
Source: Sci Rep. 2018 Sep 26;8:14429. doi: 10.1038/s41598-018-32397-z (PMC6158293; doi:10.1038/s41598-018-32397-z)

**Longitudinal Patterns in Clinical and Imaging Measurements Predict Residual Survival in Glioblastoma Patients**

Nova F. Smedley^1,2^, Benjamin M. Ellingson^1,3,4^, Timothy F. Cloughesy^4,5^, William Hsu*^1,2^

# Supplementary materials

## Supplementary methods

### Inclusion criteria

A total of 314 GBM patients were retrospectively reviewed, consisting of 9693 clinical visits and 30 644 recorded events between December 1999 and May 2015. Data was cleaned by removing events with missing information (762 events) and invalid values (14 events). The inclusion criteria were applied to select only patients with surgical resection followed by completion of standard chemoradiation, i.e., radiotherapy with concomitant temozolomide^2^. As a result, eight patients and their associated 263 events were excluded. Events prior to the chemoradiation were also excluded (2904 events). Events that included death or hospice were not apart of the sequential pattern mining, resulting in a removal of 2 patients (only had death or hospice events) and 1712 events. Subsequently, there were 304 patients, 7078 visits, and 24 989 events left for further analysis.

**Data discretization**

Sequential pattern mining operates on discrete events, where patterns exponentially explode if events have many possible definitions. Since several variables in our dataset were continuous, they were discretized in the following manner. Tumor volume information was discretized into ten states with equal frequency for each type, see Table 2. Age was discretized into decades, and was the only patient covariate that was dynamic. Adjustments were also made to ethnicity and tumor location to avoid categories that had occurred in less than 10% of patients. Given that the patient population was overwhelmingly white (71%), ethnicity was binarized into two groups: white and non-white. In addition, tumors that were diagnosed in less commonly reported locations such as the thalamus, corpus callosum, cerebellum, pineal gland, and midbrain were aggregated into a single category. Since tumor location can take on multiple values (e.g., tumors that span temporal and occipital lobes), we created a variable for each location with binary values that represent whether the tumor was observed at that location.

### Pipeline implementation

Data processing, model training and testing, and plotting were implemented in R (version 3.3.1) on an Ubuntu machine (14.04). The following packages were used: RMySQL for database queries; arules and arulessequences for all sequential pattern mining steps; caret for data partitioning and all steps of cross-validation; glm and glmnet for logistic regression and LASSO regularization; and ROCR, pROC, and MLMetrics for obtaining performance metrics and related calculations.

### cSPADE parameters

The cSPADE algorithm included several parameters: minimum support, maximum size, maximum length, minimum gap, maximum gap, and maximum window. Minimum support values fell in the range [0,1] and all others, if included, can only be a single positive integer. Of these six constraints, only four were explored in two rounds. In the first round, we explored a minimum support of from 0.05 to 0.4 by increments of 0.05; maximum gap of 15, 30, 45, or 60 days between two clinical visits; maximum length of 2 clinical visits; and maximum size of 2–7 clinical events in a clinical visit. We noticed that performance was not improved by using a maximum size of 5, 6, or 7 events nor by considering a minimum support below 0.2. Therefore, in the second round, we removed those values in our grid search and considered a maximum length of 3 clinical visits. This resulted in 252 different possible cSPADE parameter combinations.

Grid search was used to identify the optimal set of cSPADE parameters. The search was repeated for each representation of tumor volume measurement (actual value, rate of change, percent change, response criteria), resulting in a total of 1008 parameter combinations that were explored.

### Model training

For LASSO regularization, the lambda value determines the amount of sparsity, or the number of features that are selected. 1000 different lambda values were explored by setting a maximum value of 0.2 and identifying 1000 log steps, i.e., generating a sequence from log(0.0001*0.2) to log(0.2) with 1000 equal steps and then exponentiating the entire sequence.

The data was split into 75% training and 25% testing partitions. Different models were created based on the type of tumor volume information used, the set of cSPADE parameters used to generate the temporal patterns, and the value of lambda to use if LASSO was included. Therefore, within the training partition, repeated 10-fold cross-validation was used to identify the best lambda value and the best model with the highest area under the ROC curve. The process shown in **Figure S1** was performed 3 times.

The performance of the model for a particular lambda value, if included LASSO, was averaged over all 10 folds in cross-validation. Each model is assigned a lambda that resulted in the highest performance. Then, each model is compared and the model with the highest performance was selected as the final model. The final model is tested on the testing partition, which was untouched during model training and parameter selection.

### Classification

The logistic regression model outputs a probability of death for each clinical visit. To determine which class the visit should be classified as, a threshold was applied. Visits where the model’s estimates were above the threshold are classified as the “residual survival ≤ x-months” class and visits below the threshold are classified as the “residual survival > x-months” class. Each possible model estimate was used as a threshold, and each threshold has a set of performance metrics associated with it—e.g., true positive rate (sensitivity, recall), false positive rate (1−specificity), precision, and average precision (precision averaged across all recall values). The threshold is shown in **Figure 3** as the grey horizontal line; it was selected by identifying the threshold with the highest f1-score, a measure of classification accuracy. This value was selected using the training partition.

### Number of visits and patients in the final models’ training and testing partitions

The final models used tumor volume rate change as a predictor, requiring at least two observations. Subsequently, there were 367 visits where baseline volumes were measured and were not apart of the models’ dataset. Since the 2-, 6-, and 9- month used patterns generated from the same maximum length and maximum gap days cSPADE parameters, these models required visits with a prior history of 120 days. This resulted in 5166 (3626 training and 1540 testing partitions) clinical visits from 259 patients (194 testing and 65 training partitions) in the 2-, 6- and 9-month models’ datasets. Therefore, the univariate analyses were computed also using 5166 visits for the final models.

### Correlation between neurologic evaluations

Mental status is a part of the neurological examination related to attention, language, and memory. Neurologic function includes mental status and other non-mental status evaluations such as weakness, numbness, and difficulty of walking. Overall neurologic status measures the differences in neurologic function. KPS is a related evaluation, but accounts for independence. To find the correlation between two neurologic evaluations Kendall’s rank correlation in R was used. All scores were continuous values. Significant tests were two-tailed and used to test if the true correlation between two exams was 0. The most correlated pair was between KPS and neurologic function (­n = 5501 visits, correlation = −0.710, p < 0.001). The least correlated pair was mental status and overall neurologic status (n = 5200 visits, correlation = −0.142, p < 0.001). All other pairs had absolute correlations between 0.400 and 0.190 and p < 0.001.

## Supplementary figures

**Figure S1.** The model-training procedure. Models not using LASSO do not have the lambda step.

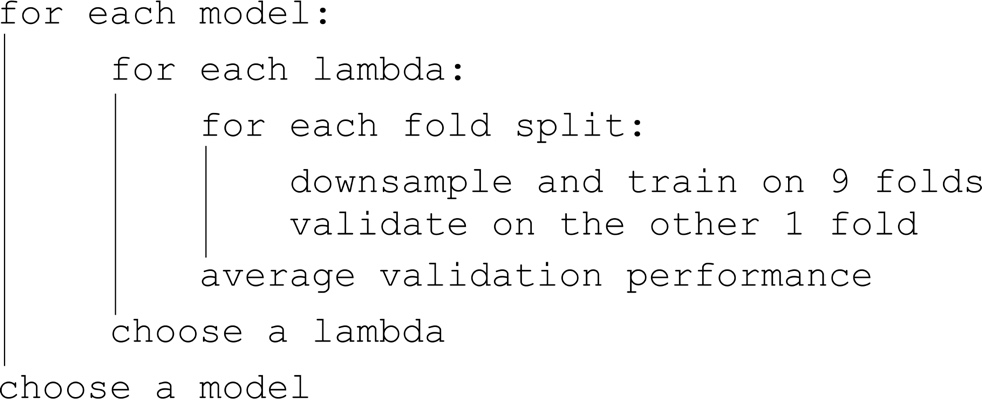


**Figure S2.** The frequency of visits in of each patient in the cohort (n=304) used for predicting patient prognosis. Tumor volume was a part of approach 1 (tumor volume) and approach 2 (tumor volume and patient covariates). Tumor volume and neurological evaluations were apart of approach 3 (temporal patterns and patient covariates), and therefore a higher visit mean. The temporal patterns visit frequencies were based on the visits ultimately used by the final model, i.e., after removing visits with insufficient history.

**
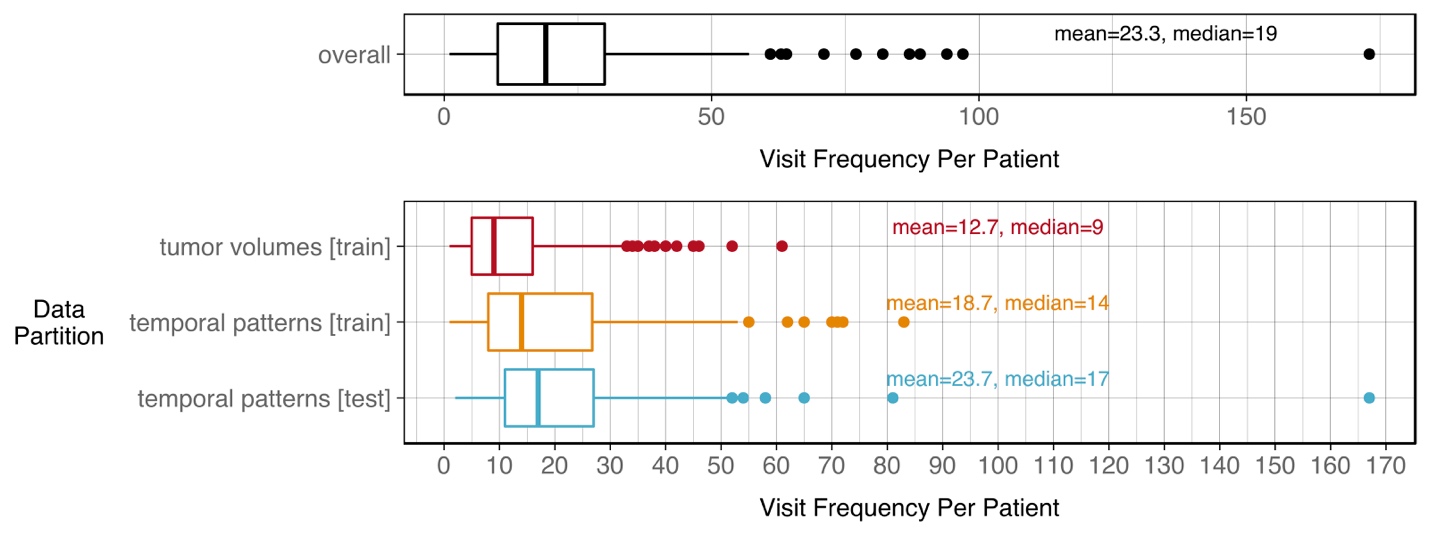
**

**Figure S3.** The frequency of each event used for sequential pattern mining.


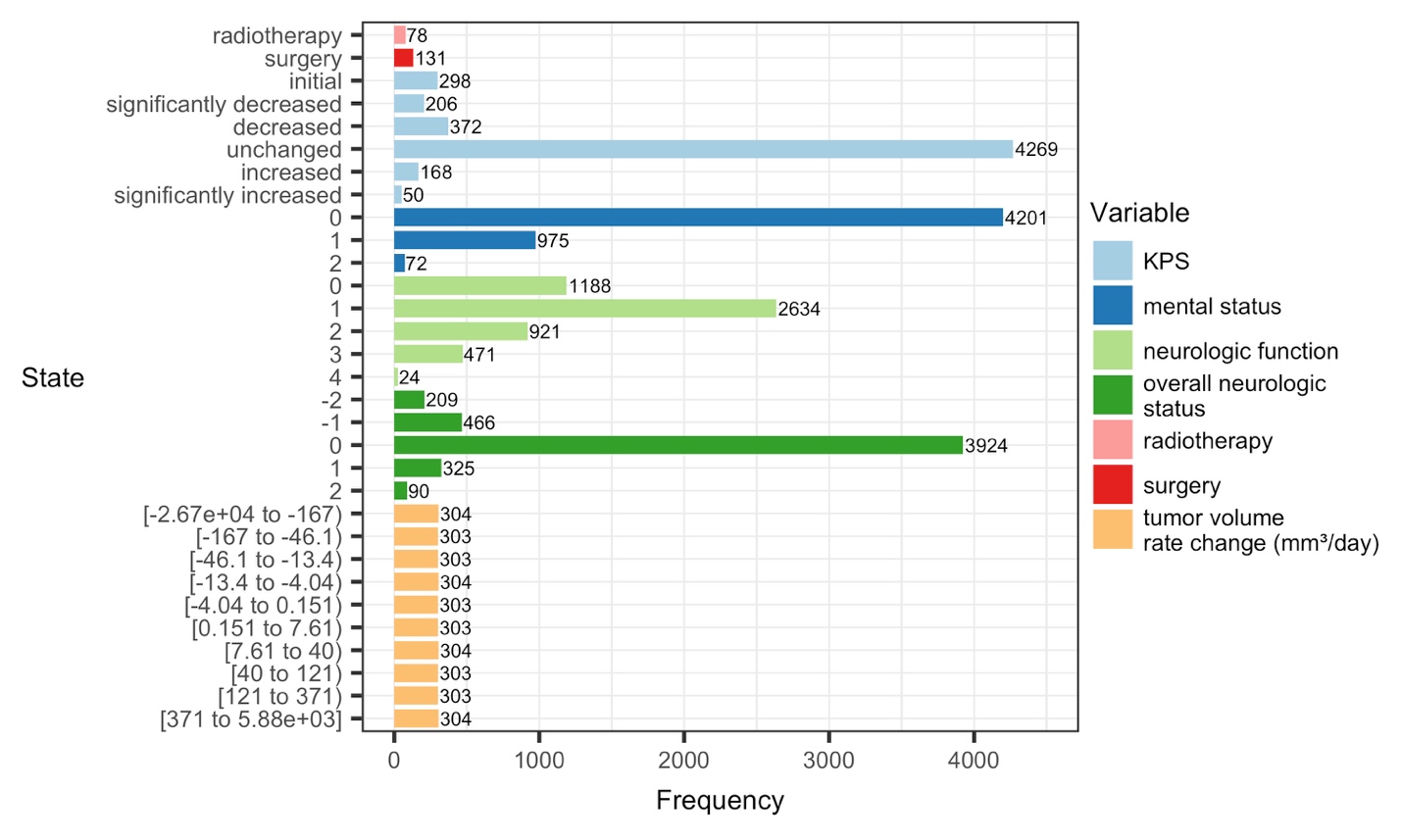


**Figure S4.** The training ROC and precision-recall curves for each type of continuous tumor volume measurement. When using tumor volume information alone to predict death in upcoming months, measurements in mm^3^ had the highest performance. The inflection and plateau points in the rate change curve represents a range of rate changes where increasing the rate change threshold resulted in more false positives while the increase in true positives was minimal (see **Figure S5**).


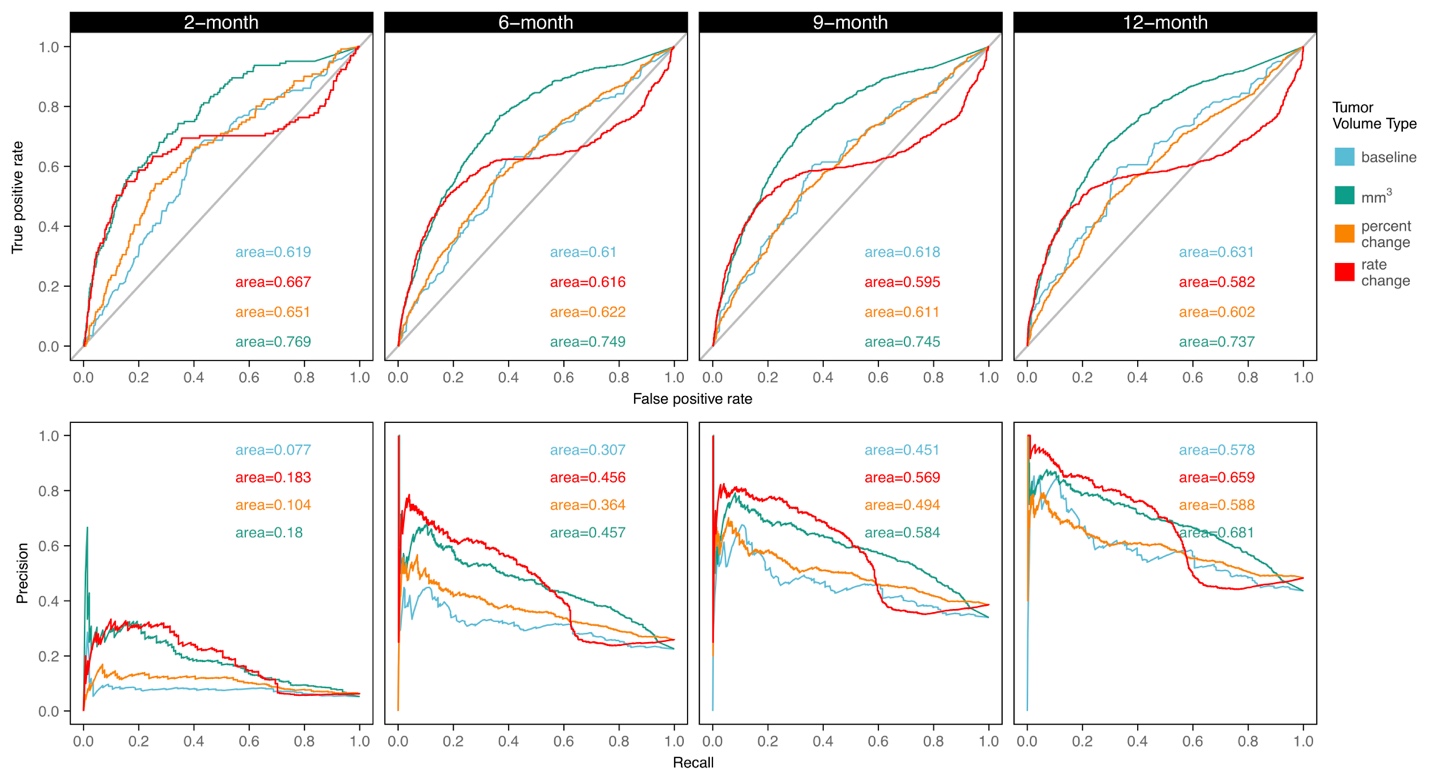


**Figure S5.** The training ROC curves for each type of continuous tumor volume measurement, i.e., rate change in mm^3^/day (A), volume in mm^3^ (B), baseline volume in mm^3^ (C), and percent change (D) for predicting patient prognosis. Each point within the ROC curve was a tumor volume measurement that was used as a threshold for classification in the training partition, where values above the threshold predicted patient residual survival ≤ x-months and all other values predicted residual survival > x-months.


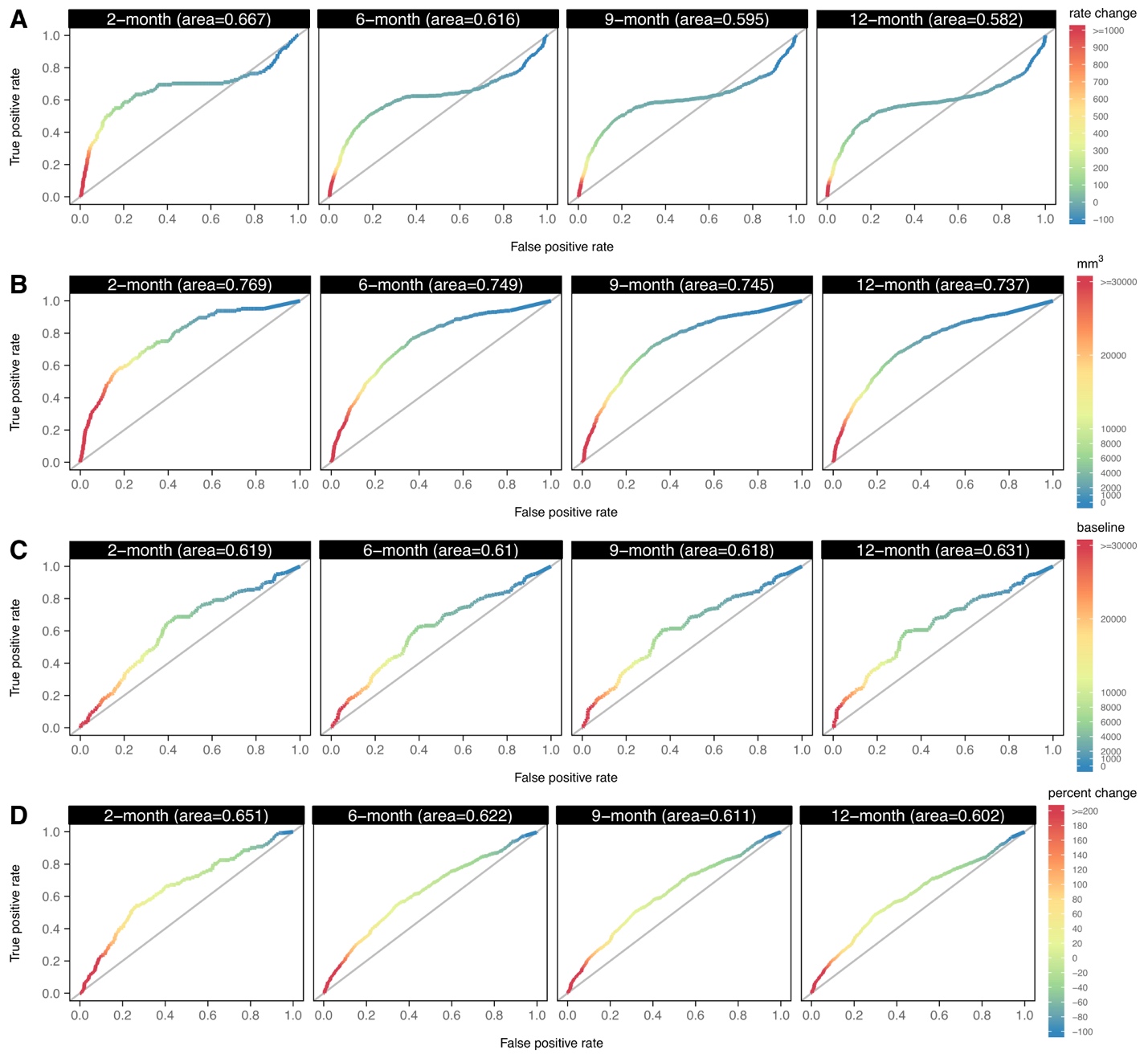


**Figure S6.** The training performance of logistic regression models when using combinations of tumor volume information (continuous values versus discretized intervals) and patient covariates (with or without) as predictors for 2-, 6-, and 9-month residual survival. Area under the ROC or PR (precision–recall) curve is averaged over each fold in 10-fold cross-validation.


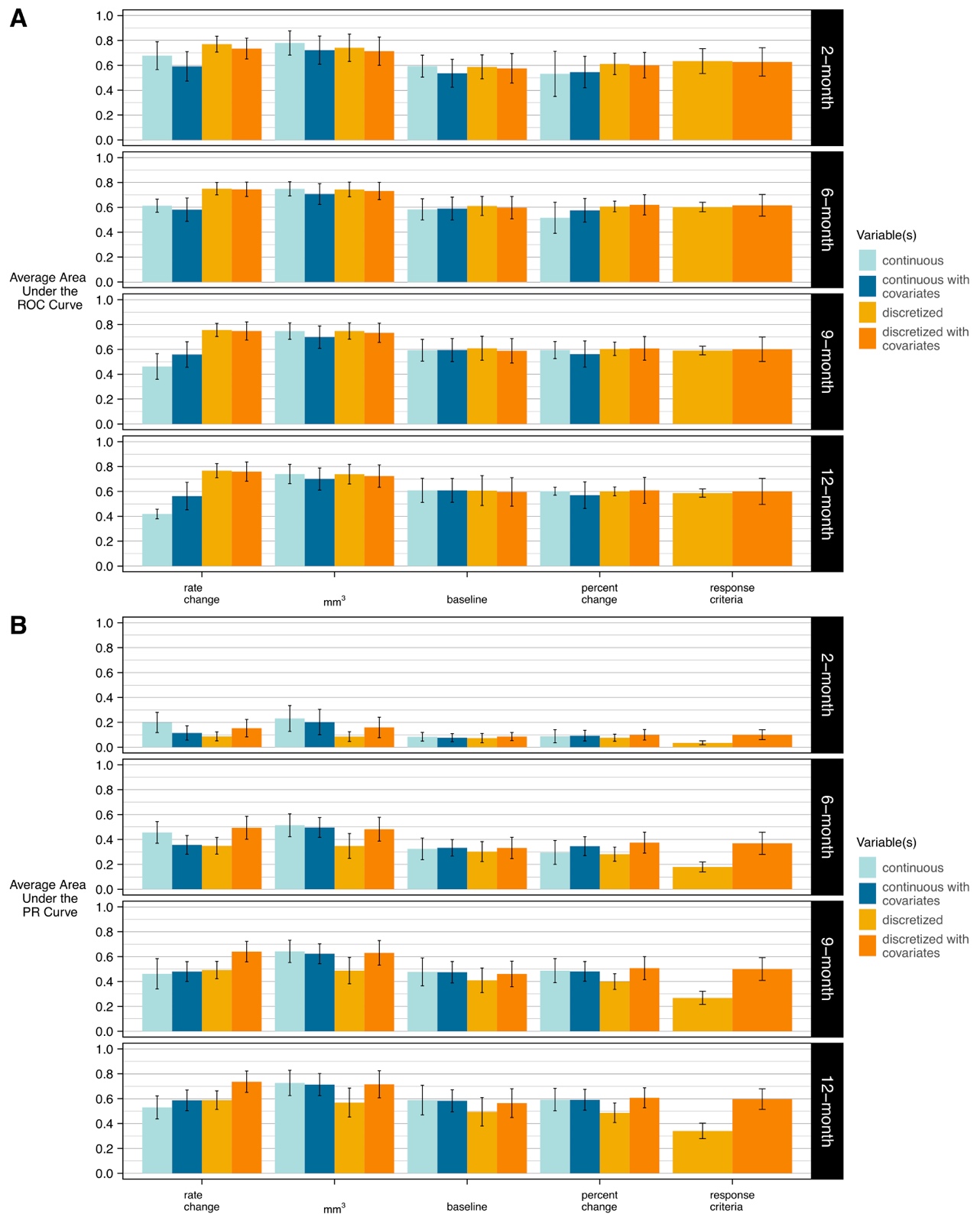


**Figure S7.** The training performance of logistic regression models when using temporal patterns and covariates as predictors for 2-, 6-, and 9-month residual survival. The top 15 sequential pattern mining constraints are shown, where the area under the ROC or PR curve is averaged over each fold in 10-fold cross-validation.


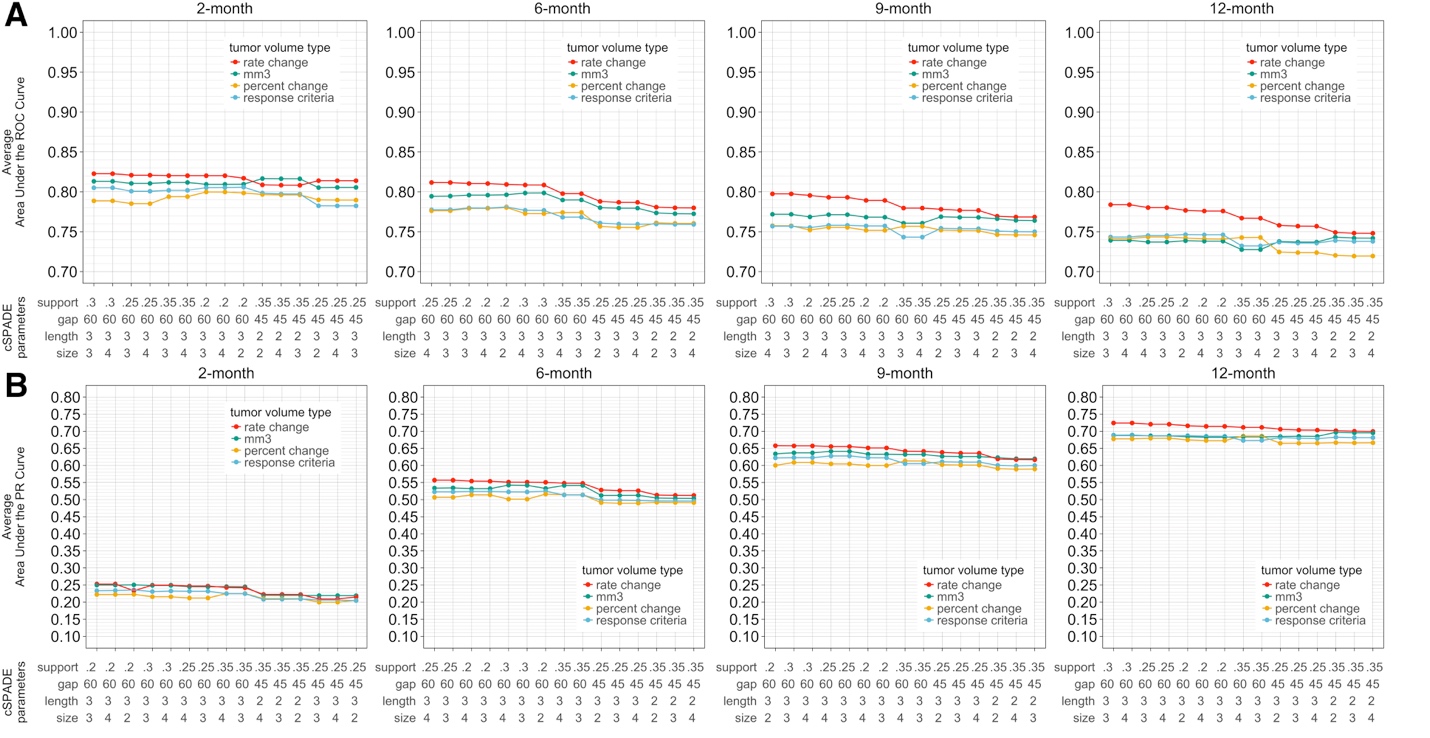


**Figure S8.** Corresponding patient events that complement **Figure 3**.


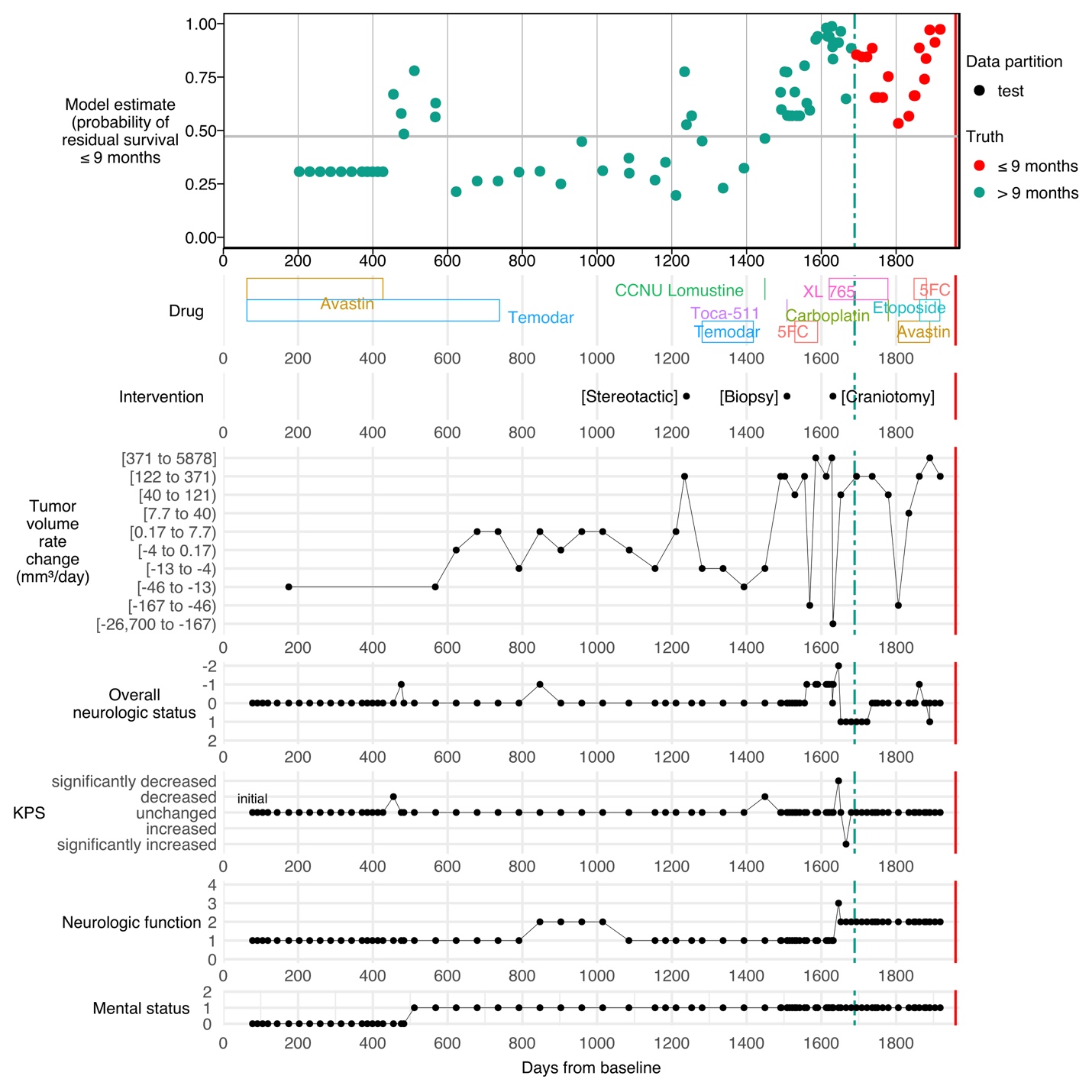


**Figure S9.**  An example of a patient (#78) where the 9-month residual survival model had *few* classification error.


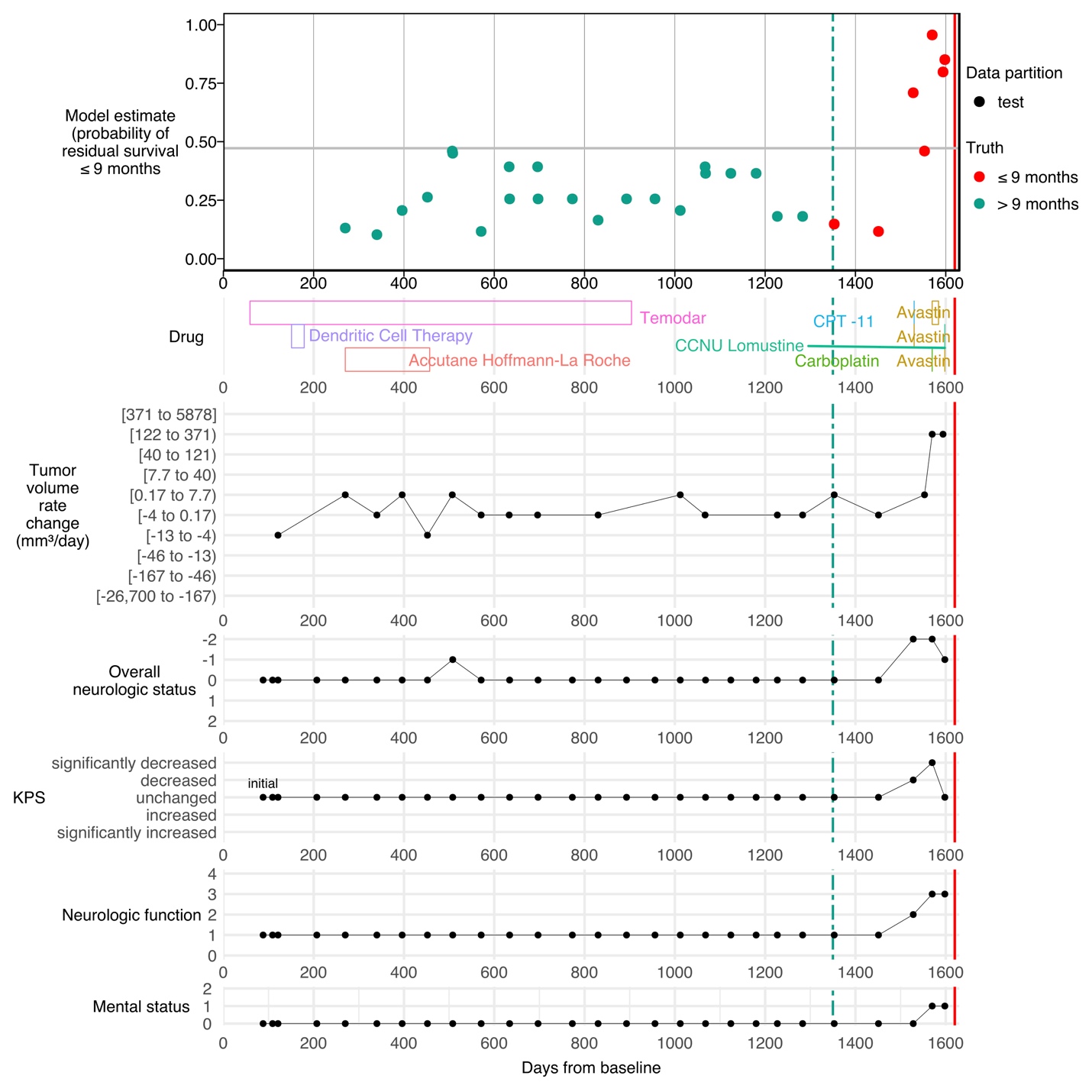


**Figure S10.**  An example of a patient (#101) where the 9-month residual survival model had *some* classification errors.


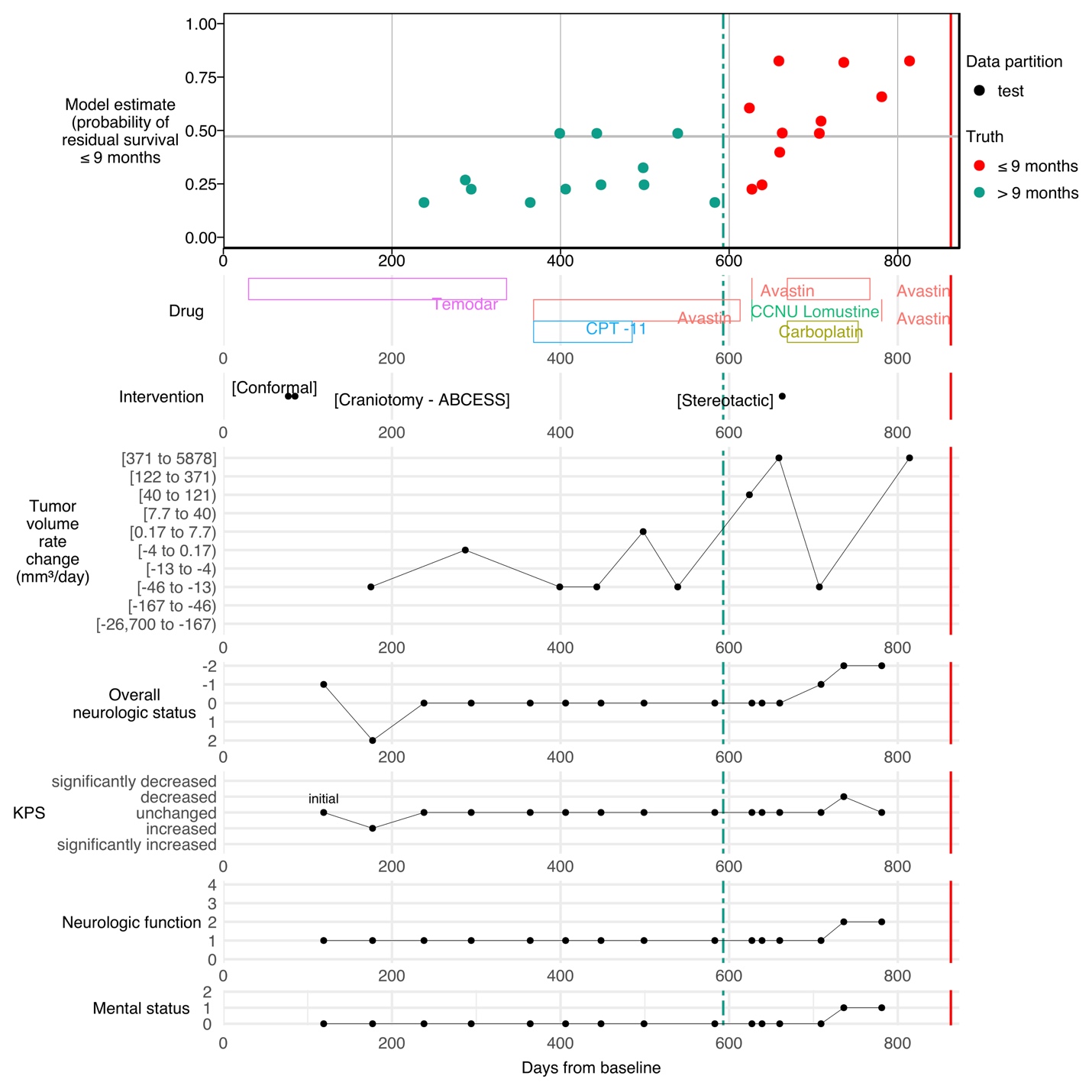


**Figure S11.** An example of a patient (#153) where the 9-month residual survival model had *many* classification errors.


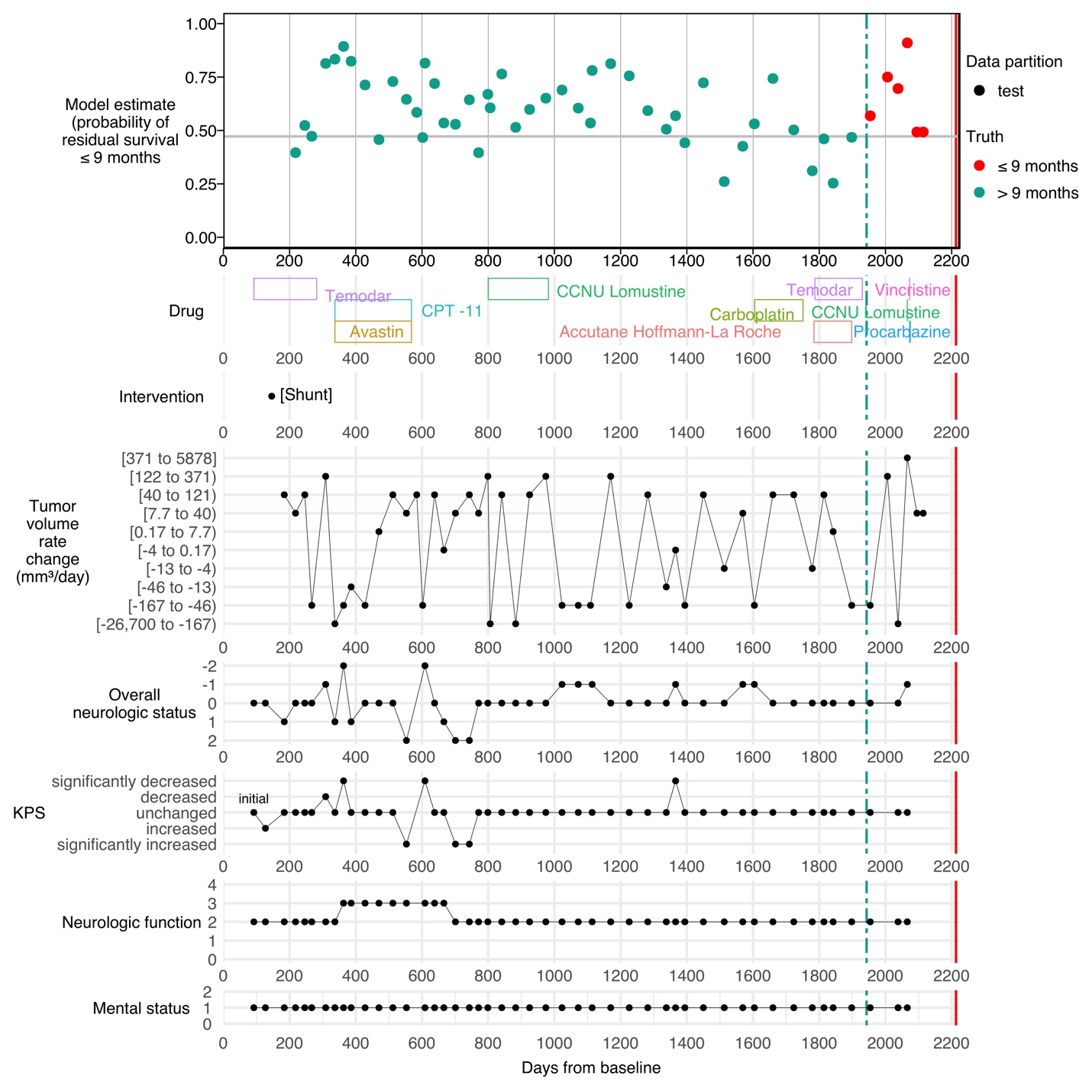

Supplement: Supplementary file 1 — Supplemental Materials [file 41598_2018_32397_MOESM1_ESM.docx]
